# Incides of C-Reactive Protein, Tumor Necrosis Factor-α, Adiponectin, Leptin and Resistin in the Blood of Patients Suffering from Chronic Pancreatitis and Type 2 Diabetes Mellitus

**DOI:** 10.25122/jml-2020-0153

**Published:** 2020

**Authors:** Dmytro Olecsandrovich Hontsariuk, Kateryna Victorivna Ferfetska, Tamara Mikolaivna Khrystych, Olexander Ivanovich Fediv, Tetjana Georgiivna Temerivska, Evelina Olexandriivna Jiguleva, Ludmila Mikchaliivna Honcharuk, Oksana Yuriivna Olinik

**Affiliations:** 1. Department of Internal Medicine and Infectious diseases, Bukovinian State Medical University, Chernivtsi, Ukraine; 2. Department of Physical Rehabilitation, Ergotherapy and Home Care, Yuriy Fedkovych Chernivtsi National University, Chernivtsi, Ukraine; 3. Department of Physical Rehabilitation and Medical and Biological Basis of Physical Education, Kamianets-Podilskyi Ivan Ohiienko National University, Kamianets-Podilskyi, Ukraine

**Keywords:** Chronic pancreatitis, type 2 diabetes mellitus, tumor necrosis factor, C-reactive protein, adiponectin, leptin, resistin., CRP – C-reactive protein, CP – cronic pancreatitis, PHP – practically healthy persons, TNF-α – Tumor necrotic factor – α

## Abstract

Studying the significance of the immune response to damage and adipokine levels is urgent regarding the development of both chronic pancreatitis and type 2 diabetes mellitus. Our objective was to study the indices of tumor necrosis factor-α (TNF-α), C-reactive protein (CRP), adiponectin, leptin, and resistin as links for triggering the mechanisms of development and progression of the low-grade chronic systemic inflammatory response in chronic pancreatitis (CP) patients with type 2 diabetes mellitus (T2DM). The study groups consisted of 87 patients: 47 patients with isolated CP (group I), 40 with CP combined with T2DM (group II), and 41 practically healthy persons. It has been established that in patients with isolated CP, the TNF-α concentration showed a reliable 1.57-fold (p <0.05) increase compared to practically healthy persons (PHP) and a 1.32-fold increase in patients who also had T2DM (p<0.05). CP patients with type 2 diabetes mellitus had the highest CRP indices (5.5-fold, p <0.05. TNF-α and C-reactive protein indices were higher in patients with chronic pancreatitis and T2DM than those with isolated chronic pancreatitis, characterizing the persistence of chronic systemic inflammation in case of the combined clinical course of these diseases.

## Introduction

Low-grade chronic systemic inflammation determines the possibility of chronic pancreatitis (CP) and type 2 diabetes mellitus (T2DM) coexistence. It depends on the intensity of the immune response to damage. During the implementation, significance is given to pro-inflammatory cytokines and CRP [1, 2]. According to some authors, C-reactive protein (CRP), as a protein of acute phase of inflammation, is a more informative marker of chronic systemic inflammation in the body [3] since it supports the inflammatory series in patients with CP, obesity, and diabetes mellitus [4].

A reliable increase of TNF-α in the blood was determined not only in the blood of patients with recurrent CP and during the period of exacerbation but also in the remission phase, which testifies to the prolongation of the chronic low-inflammatory process in the pancreas (PG) in the inter-recurrent period [5]. However, TNF-α is not only a pro-inflammatory cytokine (important in the pathogenesis of CP) but also an adipokine that plays a certain role in the development of T2DM [6].

Due to the effect of insulin in the muscles, liver, and blood vessels, adipokines are involved in the development of insulin resistance (IR) and chronic systemic inflammation in CP and T2DM. Resistin is considered to be such an adipokine. Together with TNF-α, it influences the development and progression of IR, which supports the persistence of chronic inflammation [7]. Leptin activates the macrophages, T-lymphocytes and stimulates cytokines secretion by these cells, and also correlates with CRP levels [8, 9]. In addition, an increase in the level of leptin in the blood of patients with visceral obesity and T2DM, which affects the severity of IR, has been revealed [10]. According to the data cited in the literature, leptin is also involved in the progression of chronic systemic inflammation in T2DM. In the development of IR, adiponectin’s significance is represented by its insulin-like effect. In systemic inflammation, its use is shown by the reduction of TNF-α secretion [11].

Therefore, the study of the mechanisms of chronic systemic inflammation with the participation of cytokines, CRP, adiponectin, leptin and resistin levels in patients with CP and T2DM is an urgent matter.

The aim of the research was to study the status of TNF-α, CRP, adiponectin, leptin and resistin as links for starting up mechanisms for the development and progression of the low-grade chronic inflammatory response in patients with CP and T2DM.

## Material and Methods

We examined 87 patients (47 patients had isolated CP – group I, 40 patients had CP and T2DM – group II) and 41 practically healthy persons. The age and gender of the patients did not significantly differ. The remoteness of the diseases varied from 5 to 15 years.

CRP levels were established using the TOV NVL “Granum” (Ukraine) kit using the principle of latex agglutination. Systemic inflammation was not ascertained at CRP value less than 6 mg/l; indices values greater than 6mg/l were considered to be evidence of an inflammatory reaction. TNF-α content was determined utilizing the Human TNF-α total platinum ELISA (Austria) kit. The study of leptin, adiponectin and resistin in the blood has been carried out using a set of reagents: Leptin ELISA (Diagnostics Biochem Inc., Canada), and Assay Max. Statistical processing of the obtained data was carried out on a “ViewSonic” personal computer using the standard packages of Microsoft Excel and Statistica for Windows (Stat Soft inc., USA). The mean arithmetic value (M), its mistake (m), and Student’s t-test (t) were calculated. Distinctions were considered to be reliable at p<0.05. The correlative analysis was carried out by determining the Pearson correlation coefficient. An r<0.3 was evaluated as weak, while r>0.7 was considered potent.

## Results

The values of TNF-α and CRP indices are shown in Table 1. The analysis of CRP results testified to a reliable increase of indices in patients with isolated CP in comparison with those in the PHP group (7.75±0.48mg/ml vs. 6.1±0.21 mg/ml, p<0.05, respectively). This can be explained by the presence of the chronic intoxication syndrome in patients with CP and T2DM. The highest CRP values were found in patients with CP and T2DM (40.5±1.2mg/ml that is 5.23 times higher than isolated CP, p<0.05). TNF-α indices in patients with isolated CP were reliably higher than those in the PHP group (4.51±0.17, pg/ml vs. 2.89±0.14, pg/ml, p<0.05, respectively). An increase in the given indices was more significant (1.5-times greater) in patients with a combination of CP and T2DM than patients with isolated CP (p<0.05). We have established an increase of leptin and resistin indices against the background of an adiponectin decrease in patients of both groups (Table 2).

**Table 1:** Tumor necrosis factor-α and C-reactive protein levels in the blood serum of patients with chronic pancreatitis and type 2 diabetes mellitus, M±m.

Indices	Practically healthy persons (n=41)	Chronic pancreatitis (n=47)	Chronic pancreatitis and type 2 diabetes mellitus (n=40)
**TNF-α, pg/ml**	2.89 ± 0.14	4.51 ± 0.17*	6.765 ± 0.34*/**
**CRP, mg/ml**	6.1 ± 0.21	7.75 ± 0.48*	40.5 ± 1.2*/**

CRP - C-reactive protein; TNF-α – Tumor necrosis factor- alpha; *significant difference (р<0.05) in comparison with levels of practically healthy persons; ** significant difference (р<0.05) in comparison with levels of patients with chronic pancreatitis.

**Table 2:** Leptin, adiponectin and resistin levels in the blood serum of patients with chronic pancreatitis and type 2 diabetes mellitus, M±m.

Indices	Practically healthy persons (n=41)	Chronic pancreatitis (n=47)	Chronic pancreatitis and type 2 diabetes mellitus (n=40)
**Leptin, ng/ml**	3.7±0.89	14.67±1.73 *	45.51±5.12 */**
**Adiponectin, ng/ml**	14.21±1.4	8.2±0.21 *	4.4±0.3 */**
**Resistin, ng/ml**	4.14±0.52	9.05±0.1 *	17.2±1.73 */**

* significant difference (р<0.05) in comparison with levels of practically healthy persons; ** significant difference (р<0.05) in comparison with levels of patients with chronic pancreatitis.

Analysis of the results has shown that, in the case of isolated CP, leptin values exceed the values in the PHP group by 3.96 times (p<0.05) and constituted 14.67±1.73 ng/ml vs. 3.7±0.89 ng/ml, respectively. In the group with CP and T2DM, the indices increased by 12.3 times (p<0.05) compared to those in the PHP group and were 45.51±5.12 ng/ml. This can be the evidence that leptin is involved in the IR processes and leptin-resistance in the presence of T2DM.

Resistin is referred to as an adipokine that can influence IR development. According to the results that we have obtained, the highest resistin indices were found in patients with CP and T2DM. In these patients, the values were 1.9 times greater (17.2±1.73 ng/ml, p<0.05) compared to patients with isolated CP. It should be mentioned that TNF-α values were the highest in this group of patients.

The decrease of adiponectin indices, which has been established in the research, was more significant in patients with CP and T2DM (4.4±0.3ng/ml), which is reliably lower compared to those with isolated CP (p<0.05). Some authors confirmed the regulatory role of this hormone of the adipose tissue in carbohydrate metabolism and inflammation maintenance.

## Discussion

CP progression depends upon the intensity of the immune response and damage. The anti-inflammatory cytokines (TNF-α) and CRP play a significant role as factors of persistence and prolongation of the inflammatory process in in the pancreas [12]. CRP has immune-modulating, mediatory, and transport functions. It regulates and maintains inflammatory events in patients with CP, obesity, and diabetes mellitus [3, 4, 13]. A reliable increase of its levels in the coexistence of CP and T2DM compared to PHP and patients with isolated CP may indirectly indicate the expression of the endogenous intoxication syndrome, which is one of the main mechanisms of low-grade chronic systemic inflammation.

A reliable TNF-α increase in the blood of patients with CP and its highest levels in CP and T2DM obtained in our research may be evidence of persistence and prolonged series of the inflammatory process in PG. This shows significance not only in diagnostics and estimation of the disease severity degree but in the approach and strategy of treatment of patients during the inter-recurrent period and prevention of the following exacerbation of CP (all the more in combination with T2DM).

The data obtained confirmed the activation of the immune response mechanisms and acute-phase proteins in the development of low-grade chronic systemic inflammation, both in isolated CP, and the coexistence of CP and T2DM. This may be possible because of IR, the activity of oxidized reactions with the involvement of hyperglycemia, dyslipidemia, changes in the adipokine balance, as well as due to the chronic processes of intoxication.

TNF-α is considered to be not only an anti-inflammatory cytokine but adipocytokine, which plays a certain role in the development of T2DM. It activates the hormone-dependent lipase in adipocytes, stimulates lipolysis, and decreases lipoprotein lipase activity, also influencing the lipid metabolism, contributing to the development of both diseases [14].

Due to the investigations of the last decades, the adipose tissue started to be considered as an active organ that produces biologically active substances – adipokines [15]. Adiponectin, leptin, and resistin are referred to as adipokines. Their metabolic effects are achieved through active lipolysis, insulin effects in the muscles, liver and vessels (taking part in IR development) through an auto-, para- and endocrine mechanism. The development of IR in T2DM and obesity is influenced by both resistin and TNF-α [16]. The analysis of the results obtained by us testified that resistin values were the highest in the group of patients suffering from CP and T2DM. It should be mentioned that TNF-α indices were also the highest in this group of patients, agreeing with the results of some researchers who also indicated their role in the formation of low-grade chronic systemic inflammation [17].

An increase of leptin levels in patients with CP and T2DM is 12.3 times higher compared to PHP, and this may show its active involvement not only in IR and leptin-resistance but also in the persistence of the ow-grade chronic systemic inflammation that influences the severity of the clinical course of these diseases and formation of complications.

Adiponectin is considered to reduce the level of the circulating fatty acids, activates their oxidation in the muscular tissue, liver and, as well as leptin, prevents lipid accumulation in the cells, and keeps up the normal correlation of lipoprotein complexes and triglycerides [18]. Moreover, there is the theory that adiponectin deficiency against the background of an increasing mass of adipose tissue influences IR development of the muscular tissue and liver in diabetes mellitus [19]. The results that we obtained may confirm such conclusions. Deficiency may appear because of these mechanisms, which are directed to the level decrease of glucose in the blood, TNF-α secretion reduction, but are exhausting in the combination of CP with T2DM.

Thus, determination of adiponectin, leptin and resistin levels in the combined clinical course of CP and T2DM is of clinical interest since it may show the state of compensation of the energy, lipid, and carbohydrate metabolism, and also the intensity of chronic systemic inflammation.

## Conclusions

TNF-α and C-reactive protein levels are higher in patients with chronic pancreatitis and type 2 diabetes mellitus than those with isolated chronic pancreatitis, characterizing the persistence of chronic systemic inflammation in case of the combined clinical course of these diseases. The clinical course of chronic pancreatitis and type 2 diabetes mellitus is characterized by an imbalance in the regulatory function of adipokines, leptin, resistin and adiponectin. Leptin and resistin levels are significantly increased (comparable to TNF-α and CRP levels), but adiponectin indices are decreased. A reliable increase of TNF-α, CRP, leptin, resistin levels and decreased adiponectin levels may indicate the insufficiency of the compensatory possibilities of the immune and endocrine systems against a background of low-grade chronic systemic inflammation in these patients.

## Conflict of Interest

The authors declare that there is no conflict of interest.
